# Long-term outcomes of hemiarthroplasty for complex proximal humerus fractures: a systematic review of clinical studies with minimum 10-year follow-up

**DOI:** 10.1016/j.xrrt.2025.100616

**Published:** 2025-11-19

**Authors:** Logan D. Moews, Lord J. Hyeamang, Alexander L. Hornung, Tomas F. Vega, Jacob T. Morgan, Amelia Hummel, Matthew E. Henriques, Andrew S. Bi, Nikhil N. Verma

**Affiliations:** a Division of Sports Medicine, Midwest Orthopaedics at Rush, Chicago, IL, USA; b Rush Medical College, Chicago, IL, USA

**Keywords:** Hemiarthroplasty, Fracture, Proximal humerus, Long term, Clinical outcomes, Metallic

## Abstract

**Background:**

While hemiarthroplasty (HA) has decreased in use as a surgical treatment for complex proximal humerus fractures, its long-term performance remains poorly defined. This systematic review evaluates clinical and functional outcomes and complication rates following HA with minimum 10-year follow-up.

**Methods:**

A systematic search of PubMed, Embase, and Scopus was conducted in accordance with the Preferred Reporting Items for Systematic Reviews and Meta-Analyses guidelines to identify clinical studies reporting ≥10-year outcomes of HA for acute complex (3- or 4-part, fracture dislocations, or head split) proximal humerus fractures. Included studies reported functional or patient-reported outcomes and had a minimum average follow-up of 10 years. Study quality was assessed using the Methodological Index for Non-Randomized Studies criteria.

**Results:**

Five studies encompassing 198 patients met the inclusion criteria. Mean follow-up ranged from 10.3 to 20.5 years. Postoperative Constant scores ranged from 63.9 to 82.8. Mean forward flexion ranged from 100.0° to 126.0°, and external rotation ranged from 28.0° to 46.0°. Greater tuberosity healing rates ranged from 30.0% to 91.0%, with improved clinical outcomes associated with anatomic healing at the greater tuberosity. Failure rates ranged from 0% to 29%, most commonly due to tuberosity malunion or nonunion. Glenoid erosion was reported in up to 72.7% of patients at a mean follow-up of 20.3 years. The overall complication (range, 0%-3.2%) and reoperation (range, 0%-12.9%) rates were low. Most failures were converted to reverse total shoulder arthroplasty.

**Conclusion:**

HA for the treatment of complex proximal humerus fractures yields variable long-term clinical outcomes and high rates of failure, with the majority due to greater tuberosity malunion or nonunion. Anatomic greater tuberosity healing appears to result in improved function and lower failure risk. These findings suggest limited utility of HA in the long term, supporting the need for careful patient selection and consideration of alternative surgical options such as reverse shoulder arthroplasty for treatment of complex proximal humerus fractures.

Proximal humeral fractures are a common fracture in adults, accounting for 5%-6% of fractures.^5^^,^^6^ These injuries typically result from low energy falls in elderly patients,^20^ though younger individuals may sustain them through high-energy trauma.^5^ Treatment strategies range from conservative management to surgical treatment, including open reduction and internal fixation (ORIF), hemiarthroplasty (HA), and reverse total shoulder arthroplasty (rTSA) based on age, bone quality, and fracture pattern.^17^ Thoughtful selection of treatment modality based on individual patient and fracture factors plays a key role in maximizing functional results and minimizing the risk of adverse outcome.

ORIF is often the preferred method for younger patients with sufficient bone quality and amenable fracture patterns. While historically HA was the treatment of choice for proximal humerus fractures that were unable to undergo ORIF, rTSA, with its decreased dependence on tuberosity healing for clinical success, has dramatically increased in usage in complex proximal humerus fractures in older patients, resulting in significant decreases in HA indications.^8^^,^^19^ However, in proximal humerus fractures in young patients, HA may remain a viable option, particularly when fixation is not feasible. While short-to mid-term outcomes of HA have been well studied,^9^^,^^10^ its long-term performance remains poorly defined. Key questions persist regarding the durability of implant, complication rates, and the long-term functional outcomes in different patient populations.

Prior studies, such as a retrospective review by Yahuaca et al, have examined surgeon preferences for proximal humerus fracture treatment, noting a tendency to utilize HA or rTSA in older, osteoporotic patients and patients of all ages in which fracture patterns preclude ORIF; reserving ORIF for younger individuals with substantial bone stock.^27^ However, these studies have largely focused on short- to intermediate-term outcomes and have not adequately addressed complex fracture patterns.

The objective of this systematic review is to synthesize existing evidence on the long-term (>10 years) outcomes of HA for complex proximal humeral fractures. Specifically, we aim to evaluate patient-reported outcomes (PROs), along with complication, failure, and reoperation rates, to better define the ideal patient profile for this treatment approach.

## Methods

### Literature search methodology

A comprehensive search of PubMed, Embase, and Scopus Library databases was performed in accordance with the Preferred Reporting Items for Systematic Reviews and Meta-Analyses guidelines in April 2025 and was prospectively registered with the International Prospective Register of Systematic Reviews (PROSPERO) registration (CRD:420251103637). The following search strategy was utilized: “(“hemi shoulder arthroplasty” OR “shoulder hemiarthroplasty” OR “hemi shoulder replacement”) AND ((“long-term” OR “minimum 10 years” OR “10-year follow-up” OR “≥10 years” OR “decade follow-up” OR “longitudinal” OR “late outcomes”).” The search was performed by the author (L.M.).

Studies were included if they included males and females of any age group who underwent shoulder HA for a proximal humerus fracture, presented with a mean follow-up time of 10 years or greater, reported functional outcomes or PROs, and were published between 2000 and 2025. Studies that were cadaveric or translational, included patients with revision HA of the shoulder, did not report either functional outcomes or PROs, had fewer than 10 years of mean follow-up time, or had study designs that were systematic reviews, narrative reviews, conference abstracts, technical notes, letters to editors, or meta-analyses were excluded. Two authors (L.M. and U.D.) independently screened titles, abstracts, and full article texts using the online software program Covidence (Veritas Health Innovation Ltd., Melbourne, Australia). Any disagreements were resolved with discussion leading to consensus between the 2 screening authors.

### Data extraction and quality assessment

Data items extracted from each study included demographic information such as gender and age distribution of included patients, fracture classification (both Neer classification and patterns such as head-split and fracture-dislocations), other injuries, type of HA used, concomitant procedures; as well as outcomes such as preoperative and postoperative PROs including the Constant-Murley score and visual analog pain scale, active external rotation (ER), internal rotation (IR), and forward flexion (FF), and complications, failures defined as removal of the index HA, or reoperations. Assessment of data quality for each of the non-randomized prospective and retrospective studies included in this systematic review was performed with the Methodological Index for Non-Randomized Studies (MINORS) criteria.^23^

### Statistical analysis

Pooling of data was avoided due to the high risk of bias inherent with retrospective studies and heterogeneity among included studies. A qualitative data comparison was conducted. For studies only reporting ranges, the standard deviation was approximated as the range divided by 4. Eligible studies were entered into the R statistical software (R Foundation for Statistical Computing, Vienna, Austria) to create single-arm forest plots illustrating mean postoperative Constant-Murley scores, active shoulder FF, and active shoulder ER. These forest plots served as visual aids to summarize these means.

## Results

### Search results and study quality/risk of bias

**Figure 1:**
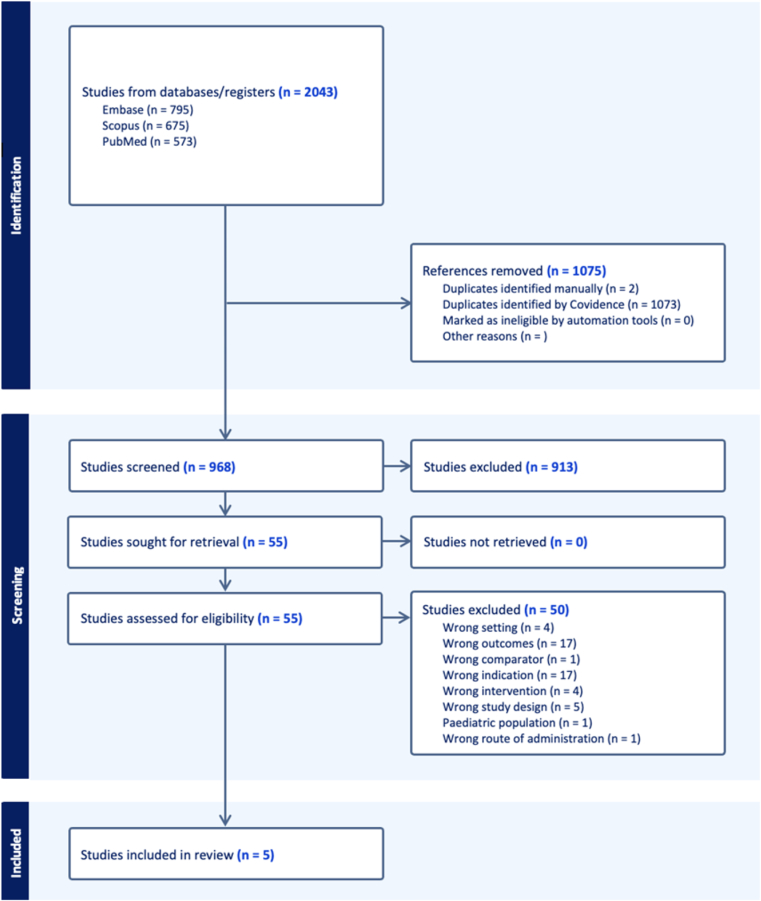
Preferred Reporting Items for Systematic Reviews and Meta-Analyses (PRISMA) study selection flow diagram. The numbers of screened, excluded, and included studies are shown.

A total of 2,043 studies were identified in the initial search, 1,075 of which were duplicates and were subsequently excluded. The remaining 968 studies underwent title and abstract screening; 913 were found to be irrelevant to the study aims and therefore excluded. The remaining 55 studies were assessed for eligibility with full-text review. After excluding 50 studies for having an incorrect study design, outcomes, comparator, indication, intervention, setting, or patient population, 5 studies^1^^,^^12^^,^^18^^,^^25^^,^^28^ were ultimately included for data extraction (Fig. 1). Each of the studies included in this systematic review evaluated functional outcomes or PROs following shoulder HA after 10 years of follow-up.

Table I summarizes study quality based on MINORS criteria for nonrandomized studies. The ideal MINORS score for noncomparative studies is 16, with scores ≤8 being the accepted cutoff for poor study quality. Each of the included studies had a score ≥9 indicating sufficiently a low risk of bias.^23^ The ideal MINORS score for comparative studies is 24, with scores ≤19 being the accepted cutoff for poor study quality. Of note, no study received points for prospective calculation of data, unbiased assessment of study endpoints, or prospective calculation of study size.

**Table I:** Summary of study quality and risk-of-bias assessment.

Study	Study design (LOE)	Quality assessment score	Sufficient study quality
Uchiyama et al,^25^ 2022	Retrospective Case Series (IV)	MINORS (noncomparative) score: 9	Yes
Hasler et al,^12^ 2024	Retrospective Case Series (IV)	MINORS (noncomparative) score: 10	Yes
Patel et al,^18^ 2024	Retrospective Case Series (IV)	MINORS (noncomparative) score: 11	Yes
Antuña et al,^1^ 2008	Retrospective Case Series (IV)	MINORS (noncomparative) score: 9	Yes
Zhao et al,^28^ 2023	Retrospective Case Series (IV)	MINORS (noncomparative) score: 9	Yes

*LOE*, level of evidence; *MINORS*, Methodological Index for Non-randomized Studies.

Each included study had a sufficiently low risk of bias.

### Demographic characteristics

In the 5 studies^1^^,^^12^^,^^18^^,^^25^^,^^28^ included in this review, there were a total of 198 patients included. There were 87 (43.9%) male patients, the mean age ranged from 28.6 to 66.0 years, and all patients had a mean follow-up time of 10.3-20.5 years. The mean time from injury to surgery, reported by 4^1^^,^^12^^,^^18^^,^^28^ of the 5 included studies, was 3.0-72.0 days. A summary of demographic characteristics for each of the studies included in this systematic review is included in Table II.

**Table II:** Summary of demographic characteristics of included studies and patient cohorts.

Cohort	N	Mean age (SD), yr	Male sex, n (%)	Mean follow-up (SD), yr	Mean time from injury to surgery (SD), d
Uchiyama et al,^25^ 2022	11	28.6 (6.9)	11 (100%)	20.5 (11)	NR
Hasler et al,^12^ 2024	31	61.2 (28.8)	21 (67.7%)	10.4 (5.5)	5 (5.3)
Patel et al,^18^ 2024	12	59.1 (7.7)	5 (41.7%)	18.7 (5.1)	72 (114)
Antuña et al,^1^ 2008	57	66.0 (28.0)	13 (22.8%)	10.3 (6.8)	3 (7.8)
Zhao et al,^28^ 2023	87	59.8 (12.2)	32 (36.8%)	14.7 (7.5)	9.5 (5.1)
Total	198				

*SD*, standard deviation; *NR*, not reported.

### Surgical indications, techniques, and arthroplasty choice

Surgical indication for HA was defined by the Neer classification of proximal humerus fractures. All studies reported injuries as complex proximal humerus fractures, which was defined as Neer 3- or 4-part fractures, fracture dislocations (n = 17), or head split fractures (n = 7). Due to the nature of injury that resulted in the complex proximal humerus fractures (car accidents, traumatic falls, etc.) within the patients included in this review, there were a variety of other reported injuries sustained by the patients.

Nearly all studies were consistent in using the same prosthesis and mode of fixation, which was a Neer, Tornier, or Cofield prosthesis that was either cemented or press-fit. There were 25 press-fit (uncemented) prostheses (12.6%) reported, and 173 prostheses were cemented (87.3%). Concomitant procedures varied across the included studies, as did the postoperative rehabilitation plans utilized. Table III summarizes surgical indication, other injuries sustained, type of prosthesis used, concomitant procedures performed, and postoperative rehabilitation plan implemented.

**Table III:** Summary of surgical indications and fixation for each included study.

Study	Surgical indication (n)	Other injuries sustained (n)	Type of prosthesis (n)	Concomitant procedures	Postoperative rehabilitation
Uchiyama et al,^25^ 2022	2-part fracture, dislocation of anatomical neck (3), 4-part fracture dislocation (4), 4-part fracture greater tuberosity (1), 3-part fracture valgus impacted (1), 3-part fracture dislocation (1)	Femoral shaft fracture (2), thoracic vertebral fracture (1), glenoid fracture (1), transient brachial plexus palsy (1), forearm fracture (1), clavicle fracture (1), transient axillary nerve palsy (1)	Neer II (10) and Aequalis CoCr (1); cemented (5), uncemented (6)	0	Immobilized for 4 weeks in internal rotation using a sling. Pendulum exercise and early passive motion were initiated on the second postoperative day. On the fifth day, passive exercises of self-assisted elevation and external rotation with a stick were added, with the patient in the supine position. Resistive exercises were added at 6-8 weeks. Stretching and isometric strengthening exercises were gradually supplemented as tolerated. Active forward elevation was allowed at 7 weeks.
Hasler et al,^12^ 2024	All complex proximal humerus fracture	NR	Zimmer (31); Cemented (20), Uncemented (11)	NR	Sling for 6 weeks and performed passive exercise in neutral rotation. Active exercise started at 6 weeks postoperatively.
Patel et al,^18^ 2024	4-part fracture (9), head splitting fracture (2), humeral shaft fracture (1)	NR	Neer II (12); Cemented (12)	NR	NR
Antuña et al,^1^ 2008	3-part fracture (7), 4-part fracture (32), 4-part fracture dislocation (9), head splitting fracture (5)	Transient brachial plexus injury (1), femoral neck fracture (1), ankle fracture (1), clavicle fracture (1), cervical spine fracture (1), glenoid rim fracture (1)	Neer II (33); Cofield (24); press-fit (8); cemented (49)	Acromioplasty (4); clavicular osteosynthesis (1)	Arm was placed in a shoulder immobilizer. The physical therapy program started with passive motion exercises within the first 48 h and during a variable period between 3 and 6 weeks, followed by a standard active-assisted motion program progressing to isometric strengthening.
Zhao et al,^28^ 2023	Complex fracture types (3-part, 4-part and/or dislocation, head splitting fracture)	Glenoid rim fracture (NR)	Zimmer, Tornier, Cemented (87)	Glenoid rim fracture anchor fixation (NR); biceps tenodesis (87)	Arm was placed in a neutral rotation brace for six weeks. Passive range of motion (ROM) exercises started at the third week after surgery. Assisted active ROM exercises were allowed six weeks after surgery. Strengthening exercises were instituted at least three months after surgery until tuberosity healing was confirmed by radiography.

*NR*, not reported.

### Patient-reported outcome measures

Due to the acuity of the injuries that resulted in complex proximal humerus fractures, preoperative PROs were routinely not reported across the studies. Of note, Hasler et al reported that 22 of the original 31 patients were available for long-term PRO follow-up.

#### Constant score and patient satisfaction

**Figure 2:**
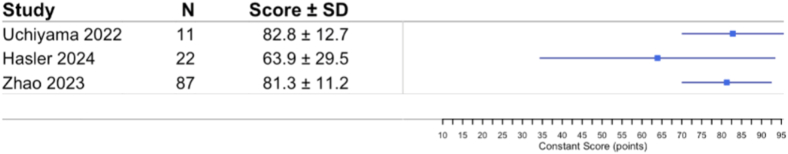
Forest plot reporting mean postoperative Constant scores across the included studies. *Black boxes* represent the mean value of each study with lines extending to the 95% confidence intervals. *SD*, standard deviation.

Constant scores were reported by 3 studies^12^^,^^25^^,^^28^ in this review. Postoperative means ranged from 63.8 to 82.8. Figure 2 displays a forest plot reporting postoperative Constant scores for the 3 studies.

Patient satisfaction data were reported by 3 studies^1^^,^^12^^,^^25^ included in this review. Uchiyama et al reported that 72.7% of their patients rated their satisfaction at either a 4/5 or 5/5 on their scale. Hasler et al reported that 63% of patients (14 of 22) rated their satisfaction as excellent, 27% (6 of 22) rated good, and 10% (2 of 22) rated fair. Antuña et al reported that 49% (28 of 57) rated their satisfaction as excellent, 28.1% (16 of 57) rated good, 3.5% (2 of 57) rated satisfactory, and 19.3% (11 of 57) rated unsatisfactory. Table IV summarizes the mean postoperative Constant scores and the patient satisfaction ratings for the included studies that reported on these outcomes.

**Table IV:** Summary of mean postoperative Constant score and patient satisfaction.

Cohort	Mean postoperative Constant score (SD)	Patient satisfaction (%)
Uchiyama et al,^25^ 2022	82.8 (12.7)	72.7 (8 of 11) rated a 4/5 or 5/5
Hasler et al,^12^ 2024	63.9 (29.5)	63% (14 of 22) rated excellent; 27% (6 of 22) rated good; 10% (2 of 22) rated fair
Patel et al,^18^ 2024	NR	NR
Antuña et al,^1^ 2008	NR	49% (28 of 57) rated excellent; 28.1% (16 of 57) rated good; 3.5% (2 of 57) rated satisfactory; 19.3% (11 of 57) rated unsatisfactory
Zhao et al,^28^ 2023	81.3 (11.2)	NR

*SD*, standard deviation; *NR*, not reported.

### Active range of motion

Preoperative range of motion (ROM), like the PROs, was generally not reported across studies due to the acute presentation of the proximal humerus fractures.

#### Forward flexion

**Figure 3:**
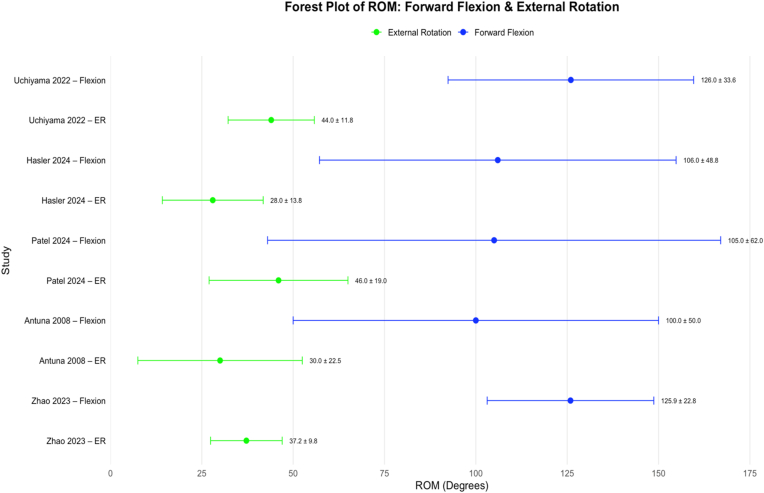
Forest plot reporting mean postoperative active forward flexion and external rotation measurements across the included studies. *Green* data points represent external rotation, and *blue* data points represent forward flexion. The *green and blue dots* represent the mean value of each study with lines extending to the 95% confidence intervals. *ROM*, range of motion; *ER*, external rotation.

Mean values of active FF were reported by all 5 studies included in this review. The mean postoperative values ranged from 100.0 to 126.0°. Figure 3 displays a forest plot reporting postoperative active FF measurements for all studies included.

#### External rotation

Mean values of active ER were reported by all 5 studies included in this review. The mean postoperative values ranged from 28.0 to 46.0°. Figure 3 displays a forest plot reporting postoperative active ER measurements for all studies included.

#### Internal rotation

Mean values of active IR were reported by all 5 studies included in this review. Four^1^^,^^12^^,^^18^^,^^25^ of the 5 studies reported IR at a spinal level; mean postoperative values ranged from L5 to T10. Zhao et al recorded the mean postoperative measurement in degrees, which was 15.7.

### Radiologic data

All 5 studies reported radiologic data at final follow-up via X-rays. Of note, Hasler et al, Antuña et al, and Zhao et al noted that they lost 9, 11, and 40 patients, respectively, for final radiologic follow-up. Greater tuberosity healing rates at final follow-up ranged from 30% to 91% across all studies. Hasler et al recorded 86.4% of patients showed evidence of lesser tuberosity healing. Rates of malunion and nonunion varied across the 2 studies^1^^,^^28^ that reported on these findings. Four^1^^,^^12^^,^^25^^,^^28^ of the studies reported findings on glenoid erosion at final follow-up which varied based on severity, and location. Patel et al and Antuña et al both reported data on radiologic evidence of prosthesis loosening and instability. Table V summarizes the radiologic findings reported by the included studies in this review.

**Table V:** Summary of radiologic findings.

Cohort	Greater tuberosity status	Lesser tuberosity healing (%)	Glenoid wear/Erosion (%)	Prosthesis loosening (%)	Radiographic subluxation (%)
Uchiyama et al,^25^ 2022	91% healed (10 of 11); 81.8% anatomically united (9 of 11); 18.1% resorbed (2 of 11)	NR	72.7 (8 of 11) grade 1; 18.0 (2 of 11) grade 2; 8.0 (1 of 11) grade 3	0	NR
Hasler et al,^12^ 2024	77.3% healed (17 of 22); 50.0% anatomically united (11 of 22); 10% resorption (2 of 20)	86.4 healed (19 of 22); 63.6 (14 of 22) anatomically united	9.0 (2 of 22)	0	NR
Patel et al,^18^ 2024	58.3% healed (7 of 12); 0% malunion; 41.7% resorbed (5 of 12)	NR	NR	8.3 (1 of 12)	25.0 (3 of 12)
Antuña et al,^1^ 2008	62.9% healed (22 of 35); 2.8% nonunion (1 of 35); 8.6% malunion (3 of 35); 25.7% resorption (9 of 35)	NR	22.9 mild (8 of 35); 11.4 moderate 94 of 35); 2.9 severe (1 of 35)	2.8 (1 of 35)	74.3 (26 of 35)
Zhao et al,^28^ 2023	34.4% healed (30 of 87); 11.5% malunion/nonunion (10 of 87); 10.3% (9 of 87) resorption	NR	56.8 concentric (21 of 37); 8.1 eccentric (3 of 37)	NR	NR

*NR*, not reported.

### Failures, complications, and reoperations

Among the studies reviewed, there was substantial variability in reported rates of failure, complications, and reoperations, highlighting differences in patient populations, surgical techniques, and follow-up durations. Uchiyama reported a 0% failure, complication, and reoperation rate. Hasler et al reported a 29.0% (9 of 31) failure rate; 5 were due to malunion, displacement, or nonunion of the tuberosities within the first 2 years postoperatively, and 2 were due to rotator cuff insufficiency after 6 and 7 years. They were all converted to rTSA. The 2 other failures recorded had a *Cutibacterium acnes* infection that was treated with a 2-stage surgery (implant removal and spacer insertion and rTSA implantation after antibiotic therapy). Hasler et al reported 4 reoperations, which were an L'Episcopo tendon transfer for pseudoparalysis of ER, 2 patients with frozen shoulder that required arthroscopic capsulotomy at 12 and 14 months postoperatively, and 1 periprosthetic infection that required an open débridement without the need for HA removal. Hasler et al reported 1 other complication which was a periprosthetic infection that was treated with antibiotics. Patel et al reported a failure rate of 16.7% (2 of 12) in which they were converted to rTSA for post-traumatic arthritis and rotator cuff failure at 12.5 and 13.0 years after the index HA, respectively. Antuña et al reported a failure rate of 3.5% (2 of 57). One patient required a prosthesis removal and shoulder fusion 6 years after the index surgery due to severe limited ROM; this same patient underwent a greater tuberosity osteotomy and rotator cuff repair shortly after the index procedure and a pectoralis major transfer at 9 months postoperatively. The other failure was due to symptomatic humeral component loosening and glenoid arthritis at 11 years after the index HA, and this patient was converted to an anatomic total shoulder arthroplasty. Zhao et al reported a failure rate of 2.3% (2 of 87), in which both failures were converted to rTSA due to deep infection within 2 years after the index procedure. They noted 1 other reoperation which was multiple débridement procedures due to infection at 4 years postoperatively. Zhao et al reported one other complication which was an anterior dislocation due to severe glenoid erosion at 5 years postoperatively.

## Discussion

The results of this systematic review indicate that HA for the treatment of complex proximal humerus fractures results in suboptimal healing and high failure rates (0%-29%). Our review found that while HA may provide adequate pain relief for some patients, the incidence of failure and dissatisfaction remains high, and clinical and functional outcomes vary significantly. These results highlight the need for careful patient selection and consideration of alternative treatment strategies to optimize outcomes for complex proximal humerus fractures.

Although only 3 of the included studies^12^^,^^25^^,^^28^ reported Constant scores, the postoperative values varied significantly at the final follow-up. In the absence of preoperative values, evaluating the change in Constant scores was not feasible, limiting the ability to fully assess the clinical impact of the HA procedure. Achieving proper reduction of the greater tuberosity is a crucial aspect of the HA procedure, as it has been reported by Lazzarini et al that anatomically reduced greater tuberosities had significantly improved University of California Los Angeles scores compared to nonanatomically reduced tuberosities, as observed at the 2-year final follow-up.^16^ This notion is supported by the results of this review, as Hasler et al reported only 50% of patients showed evidence of anatomic reduction while also reporting the lowest mean Constant scores postoperatively.^12^ Of note, Hasler et al excluded the 9 revision cases from their PRO follow-up data collection. Uchiyama et al and Zhao et al reported the highest rate of anatomic union, 81.8% and 89.7%, respectively, with both studies demonstrating relatively high postoperative Constant scores.^25^^,^^28^ Uchiyama et al also reported a mean postoperative Japanese Orthopaedic Association score of 87.9. Thus, at long-term follow-up, achieving anatomic union of the greater tuberosity remains a critical factor in the success of HA. The aforementioned evidence underscores the importance of meticulous surgical technique to ensure optimal healing and long-term success of ORIF. This inconsistency in anatomic reproduction and healing is likely responsible for high variability in patient outcomes.

Regarding the failure rates reported in this systematic review, Uchiyama et al reported a 0% failure, complication, and reoperation rate. As previously noted, they also reported high rates of healing and anatomic union on radiologic follow-up. This suggests that not only does proper anatomic reduction correlate with improved PRO scores, but it may also influence postoperative failure rates. This may also relate to their indications for HA vs. nonoperative treatment, ORIF, or rTSA. This is further supported by Hasler et al, who reported the highest failure rate in this review (29.0%), with the most common cause being malunion or nonunion of the greater tuberosity prior to 2 years postoperatively. They performed a subgroup analysis and identified a significant difference in nonunion rates between the failed and nonfailed groups. Hackett Jr et al reported that in their database of 359 HA revisions, 43% were due to malunion and nonunion following proximal humerus fractures.^11^ Patel et al conducted a subset analysis comparing outcomes of patients that demonstrated greater tuberosity healing to those that did not. They found statistically significant differences in FF and ER measurements, American Shoulder and Elbow Surgeons scores, and Simple Shoulder Test scores. Additionally, the 2 revisions recorded by Patel et al were in the nonhealing group. Collectively, these findings highlight the important role of proper greater tuberosity reduction in reducing postoperative failure rates.

Glenoid arthrosis, a recognized complication following shoulder HA, was another frequently cited reason for revision.^13^ Rates and the degree of glenoid erosion reported in this review varied; overall rates ranged from 9.0% to 72.7%, underscoring the risk of glenoid erosion after HA. Unfortunately, no studies reported preoperative assessments of glenohumeral arthritis, preventing further investigation of glenoid arthrosis. Moreover, Antuña et al and Patel et al reported high rates (76.0% and 25.0%, respectively) of radiographic evidence of humeral subluxation based on the degree of prosthesis translation relative to the glenoid, though only one patient from Antuña et al reported subjective feelings of instability, while none from Patel et al Antuña reported that 18 patients had isolated superior subluxation that were mild in 6 patients, moderate in 8, and severe in 4 patients; they also noted that 6 shoulders had isolated anterior subluxation which was mild in 4, and moderate in 2, and finally 2 shoulders had posterior subluxation. Patel et al reported 2 patients with evidence of inferior subluxation, and 1 with anterior subluxation. Despite the high rates of subluxation observed on radiographs, it appears to have no clinically significant effects. Overall, the clinically significant complication and reoperation rates were low, but the failure rates, defined as removal of the index prosthesis, were high.

Historically, ORIF has been considered the gold standard for treating proximal humerus fractures in young patients with adequate bone quality.^3^,^24^ Robinson et al reported median 10.8 year outcomes after ORIF for proximal humerus fractures with a mean age of 55.3 years: 77.2% had tuberosity involvement, 54.1% had complete head-shaft disengagement, and 44.0% had a dislocated head.^21^ Out of the 368 included patients, they reported a 6.8% failure rate (25 of 368), in which all were due to nonunion.^21^ This rate is markedly lower than the rates documented in this review, where higher occurrences were consistently reported; however, it appears that greater tuberosity malunion and nonunion is a common cause of failure, similar to the data in our review. Postoperative stiffness, affecting 23.6% of the population, was another complication reported that required reoperation but did not necessitate revision. In the absence of systematic reviews directly comparing long-term outcomes of HA and ORIF for complex proximal humerus fractures, so the comparisons must be considered within the limitations of this review.

Recently, rTSA has emerged as a popular option for managing complex proximal humerus fractures. A 2024 multicenter randomized controlled trial by Watts et al compared outcomes in 18 patients treated with rTSA and 18 patients treated with HA for three- or four-part proximal humerus fractures.^26^ The study demonstrated a significant difference in postoperative Constant scores favoring rTSA (51.1 ± 14.9) over HA (35.0 ± 13.5; *P* = .004) at two years of follow-up. Notably, both groups had one reported revision. These findings align with other randomized controlled trials that have shown superior clinical outcomes with rTSA in the treatment of complex proximal humerus fractures, although failure rates appear to be comparable between the 2 procedures.^15^^,^^22^ However, when assessing the greater tuberosity resorption after rTSA, a 2025 systematic review and meta-analysis of 21 studies reported a pooled greater tuberosity nonhealing rate of 31.9% (range, 15.0%-63.2%) with a mean follow-up range of 14.4-84 months.^7^ Di Naro et al similarly documented a revision rate ranging from 0% to 9%, with most revisions attributable to deep infections and none resulting from greater tuberosity malunion or nonunion.^7^ This suggests that postoperative rates of greater tuberosity complications are similar between rTSA and HA. However, the clinical impact of greater tuberosity resorption and glenoid erosion appears to be more pronounced in HA, often leading to a higher indication for revision. This may be explained by the biomechanical advantage of rTSA over HA, as rTSA allows for elevation and abduction of the arm primarily through the deltoid muscle, thereby reducing reliance on an intact rotator cuff.^7^ However, this does not imply that the greater tuberosities can simply be resected during rTSA, as evidence indicates that patients who undergo greater tuberosity repair during rTSA for complex proximal humerus fractures achieve better ROM and clinical outcomes.^14^ Regrettably, no studies to date have directly compared the long-term outcomes of HA and rTSA for complex proximal humerus fractures; therefore, these conclusions should be interpreted within the context of this study's limitations. The long-term data synthesized in this review primarily reflect HA techniques and implant designs from the 1990s through the early 2010s, before the widespread adoption of fracture-specific stems, improved tuberosity fixation methods, and rTSA as a standard treatment. As surgical techniques and implant designs have evolved, outcomes following contemporary arthroplasty are likely more favorable. Nonetheless, the historical perspective provided by these long-term HA cohorts remains valuable for understanding implant survivorship and guiding the management of patients who continue to present with these reconstructions in long-term follow-up.

Finally, while ORIF remains the most commonly used treatment for proximal humerus fractures, HA is indicated in specific cases, particularly for younger patients with complex fracture patterns that preclude successful reconstruction with ORIF.^4^ Uchiyama et al was the only study that reported mean ages less than 50 years; specifically, the mean age recorded was 28.6 years. Although the sample size was the smallest of the studies included (n = 11), the outcomes and failure rates reported with HA were favorable. Furthermore, Antuña et al reported the greatest mean age (66.0 ± 28.0 years) and the lowest patient satisfaction (19% unsatisfied). Since 4 of the 5 studies included in this review reported mean ages of >55, the overall poor clinical outcomes found in this review could be influenced by the patient age, especially since bone quality is generally poorer in elderly patients, and the risk of complications at the greater tuberosity increases.^2^ Although only 1 of 5 studies included younger patients, the results of this review provide evidence for the utilization of HA when treating complex proximal humerus fractures in younger patients.

Overall, the data presented in this systematic review underscore the limited effectiveness of HA in treating complex proximal humerus fractures, particularly when compared to alternative approaches such as rTSA, which has been shown to yield superior clinical outcomes and carries a lower risk of revision surgery. Regardless of the fixation technique employed, achieving anatomic reduction of the greater tuberosity emerges as a critical factor in minimizing the risk of revision at long-term follow-up, further emphasizing the importance of precision in surgical technique to optimize patient outcomes. Moreover, this study highlights several avenues for advancing our understanding of the utility of HA for treating complex proximal fractures. First, additional prospective studies would help minimize bias and enhance the reliability of data reporting. Furthermore, comparative research on the long-term outcomes of HA, rTSA, and ORIF could provide clearer insights into the optimal indications for each treatment, ultimately helping to define the most effective approach for complex proximal humerus fractures.

### Limitations

Several limitations are inherent to this systematic review. Most included studies used retrospective designs, introducing potential selection and reporting biases. While the methodological quality was moderate to high based on MINORS criteria, the lack of prospective, randomized trials, inherently, limits the strength of the conclusions. Importantly, none of the included studies provided a direct comparison between HA and other shoulder arthroplasty techniques, precluding definitive conclusions regarding the superiority or equivalence of HA. A key limitation of this review is the small number of eligible studies, which reflects the overall paucity of long-term data on HA for complex proximal humerus fractures. Finally, the requirement for long-term follow-up led to attrition due to patient death or loss to follow-up, reducing the overall sample size and statistical power.

Moreover, there was considerable heterogeneity across studies in surgical technique and rehabilitation protocols. Additionally, studies did not report objective measures of adherence to the prescribed rehabilitation regimen, which may affect the validity of the outcome under the intended postoperative protocol. Collectively, these limitations underscore the need for high-quality, prospective comparative trials with standardized outcome definitions to further elucidate the utility of HA for complex proximal humerus fractures.

## Conclusions

HA for the treatment of complex proximal humerus fractures yields variable long-term clinical outcomes and high rates of failure, with the majority due to greater tuberosity malunion or nonunion. Anatomic greater tuberosity healing appears to result in improved function and lower failure risk. These findings suggest limited utility of HA in the long term, supporting the need for careful patient selection and consideration of alternative surgical options such as reverse shoulder arthroplasty for treatment of complex proximal humerus fractures.

## Disclaimers

Funding: No funding was disclosed by the authors.

Conflicts of interest: Nikhil N. Verma reports the following relationships: Stryker Corporation—Intellectual property royalties (IP Royalties), other professional activities; TETRUS—Stock ownership; MLB Team Physician Society—Board of Directors or committee member (self). The other authors, their immediate families, and any research foundations with which they are affiliated have not received any financial payments or other benefits from any commercial entity related to the subject of this article.

## References

[1] Antuña S.A., Sperling J.W., Cofield R.H. (2008). Shoulder hemiarthroplasty for acute fractures of the proximal humerus: a minimum five-year follow-up. J Shoulder Elbow Surg.

[2] Baker H.P., Gutbrod J., Cahill M., Shi L. (2023). Optimal treatment of proximal humeral fractures in the elderly: risks and management challenges. Orthop Res Rev.

[3] Baker H.P., Gutbrod J., Strelzow J.A., Maassen N.H., Shi L. (2022). Management of proximal humerus fractures in adults—A scoping review. J Clin Med.

[4] Chambers L., Dines J.S., Lorich D.G., Dines D.M. (2013). Hemiarthroplasty for proximal humerus fractures. Curr Rev Musculoskelet Med.

[5] Court-Brown C.M., Caesar B. (2006). Epidemiology of adult fractures: a review. Injury.

[6] Court-Brown C.M., McQueen M.M. (2002). The relationship between fractures and increasing age with reference to the proximal humerus. Curr Orthop.

[7] Di Naro C., Costa G.G., Zocco G., Sicurella M., Testa G., Pavone V. (2025). Anatomic healing of greater tuberosity improves range of motion and functional outcomes after reverse total shoulder arthroplasty for proximal humerus fractures: an updated systematic review and meta-analysis on 21 studies. J Shoulder Elbow Surg.

[8] Dillon M.T., Prentice H.A., Burfeind W.E., Chan P.H., Navarro R.A. (2019). The increasing role of reverse total shoulder arthroplasty in the treatment of proximal humerus fractures. Injury.

[9] Fucentese S.F., Sutter R., Wolfensperger F., Jost B., Gerber C. (2014). Large metaphyseal volume hemiprostheses for complex fractures of the proximal humerus. J Shoulder Elbow Surg.

[10] Grönhagen C.M., Abbaszadegan H., Révay S.A., Adolphson P.Y. (2007). Medium-term results after primary hemiarthroplasty for comminute proximal humerus fractures: a study of 46 patients followed up for an average of 4.4 years. J Shoulder Elbow Surg.

[11] Hackett D.J., Hsu J.E., Matsen F.A. (2018). Primary shoulder hemiarthroplasty: what can be learned from 359 cases that were surgically revised?. Clin Orthop Relat Res.

[12] Hasler A., Ker A., Grubhofer F., El Nashar R., Ernstbrunner L., Gerber C. (2024). Clinical and radiographic long-term outcomes of hemiarthroplasty for complex proximal humeral fractures. J Shoulder Elbow Surg.

[13] Herschel R., Wieser K., Morrey M.E., Ramos C.H., Gerber C., Meyer D.C. (2017). Risk factors for glenoid erosion in patients with shoulder hemiarthroplasty: an analysis of 118 cases. J Shoulder Elbow Surg.

[14] Jain N.P., Mannan S.S., Dharmarajan R., Rangan A. (2019). Tuberosity healing after reverse shoulder arthroplasty for complex proximal humeral fractures in elderly patients—Does it improve outcomes? A systematic review and meta-analysis. J Shoulder Elbow Surg.

[15] Jonsson E.Ö., Ekholm C., Salomonsson B., Demir Y., Olerud P., Etzner M. (2021). Reverse total shoulder arthroplasty provides better shoulder function than hemiarthroplasty for displaced 3- and 4-part proximal humeral fractures in patients aged 70 years or older: a multicenter randomized controlled trial. J Shoulder Elbow Surg.

[16] Lazzarini F., Distefano M., Shen T., Secci G., Cresci M., Tucci R. (2024). Anatomic reduction of greater tuberosity fragment for shoulder hemiarthroplasty: a predictor of good clinical outcome. Arch Bone Jt Surg.

[17] Maier D., Jaeger M., Izadpanah K., Strohm P.C., Suedkamp N.P. (2014). Proximal humeral fracture treatment in adults. J Bone Joint Surg Am.

[18] Patel A.V., White C.A., Cirino C.M., Li T., Gross B.D., Parsons B.O. (2024). Hemiarthroplasty for proximal humerus fractures: clinical and radiographic outcomes after an average of 19 years. Semin Arthroplasty JSES.

[19] Rajaee S.S., Yalamanchili D., Noori N., Debbi E., Mirocha J., Lin C.A. (2017). Increasing use of reverse total shoulder arthroplasty for proximal humerus fractures in elderly patients. Orthopedics.

[20] Rangan A., Handoll H., Brealey S., Jefferson L., Keding A., Martin B.C. (2015). Surgical vs nonsurgical treatment of adults with displaced fractures of the proximal humerus: the PROFHER randomized clinical trial. JAMA.

[21] Robinson C.M., Stirling P.H.C., Goudie E.B., MacDonald D.J., Strelzow J.A. (2019). Complications and long-term outcomes of open reduction and plate fixation of proximal humeral fractures. J Bone Joint Surg Am.

[22] Sebastiá-Forcada E., Cebrián-Gómez R., Lizaur-Utrilla A., Gil-Guillén V. (2014). Reverse shoulder arthroplasty versus hemiarthroplasty for acute proximal humeral fractures. A blinded, randomized, controlled, prospective study. J Shoulder Elbow Surg.

[23] Slim K., Nini E., Forestier D., Kwiatkowski F., Panis Y., Chipponi J. (2003). Methodological index for non-randomized studies (*MINORS*): development and validation of a new instrument. ANZ J Surg.

[24] Solberg B.D., Moon C.N., Franco D.P., Paiement G.D. (2009). Surgical treatment of three and four-part proximal humeral fractures. J Bone Joint Surg Am.

[25] Uchiyama Y., Handa A., Shimpuku E., Imai T., Takatori N., Wasai S. (2022). Primary stemmed shoulder hemiarthroplasty for proximal humerus fractures in active patients aged ≤40 years: average 20 years of follow-up. Semin Arthroplasty JSES.

[26] Watts A.C., Jenkins C.W., Boyle S.P., Crowther M.A.A., Monga P., Packham I.N. (2024). Superior functional outcome following reverse shoulder arthroplasty compared to hemiarthroplasty for displaced three- and four-part fractures in patients 65 and older: results from a prospective multicenter randomized controlled trial - the shoulder hemiarthroplasty or reverse polarity arthoplasty (SHeRPA) trial. J Shoulder Elbow Surg.

[27] Yahuaca B.I., Simon P., Christmas K.N., Patel S., Gorman R.A., Mighell M.A. (2020). Acute surgical management of proximal humerus fractures: ORIF vs. hemiarthroplasty vs. reverse shoulder arthroplasty. J Shoulder Elbow Surg.

[28] Zhao Y., Zhu Y., Lu Y., Li F., Jiang C. (2023). Long-term outcomes of shoulder hemiarthroplasty for acute proximal humeral fractures. Int Orthop.

